# Bispyridinium Compounds Inhibit Both Muscle and Neuronal Nicotinic Acetylcholine Receptors in Human Cell Lines

**DOI:** 10.1371/journal.pone.0135811

**Published:** 2015-08-14

**Authors:** Avi Ring, Bjorn Oddvar Strom, Simon R. Turner, Christopher M. Timperley, Michael Bird, A. Christopher Green, John E. Chad, Franz Worek, John E. H. Tattersall

**Affiliations:** 1 Norwegian Defence Research Establishment, Kjeller, Norway; 2 Dstl Porton Down, Salisbury, Wiltshire, United Kingdom; 3 Institute for Life Sciences, University of Southampton, Southampton, United Kingdom; 4 Bundeswehr Institute of Pharmacology and Toxicology, Munich, Germany; University of São Paulo, BRAZIL

## Abstract

Standard treatment of poisoning by organophosphorus anticholinesterases uses atropine to reduce the muscarinic effects of acetylcholine accumulation and oximes to reactivate acetylcholinesterase (the effectiveness of which depends on the specific anticholinesterase), but does not directly address the nicotinic effects of poisoning. Bispyridinium molecules which act as noncompetitive antagonists at nicotinic acetylcholine receptors have been identified as promising compounds and one has been shown to improve survival following organophosphorus poisoning in guinea-pigs. Here, we have investigated the structural requirements for antagonism and compared inhibitory potency of these compounds at muscle and neuronal nicotinic receptors and acetylcholinesterase. A series of compounds was synthesised, in which the length of the polymethylene linker between the two pyridinium moieties was increased sequentially from one to ten carbon atoms. Their effects on nicotinic receptor-mediated calcium responses were tested in muscle-derived (CN21) and neuronal (SH-SY5Y) cells. Their ability to inhibit acetylcholinesterase activity was tested using human erythrocyte ghosts. In both cell lines, the nicotinic response was inhibited in a dose-dependent manner and the inhibitory potency of the compounds increased with greater linker length between the two pyridinium moieties, as did their inhibitory potency for human acetylcholinesterase activity *in vitro*. These results demonstrate that bispyridinium compounds inhibit both neuronal and muscle nicotinic receptors and that their potency depends on the length of the hydrocarbon chain linking the two pyridinium moieties. Knowledge of structure-activity relationships will aid the optimisation of molecular structures for therapeutic use against the nicotinic effects of organophosphorus poisoning.

## Introduction

The acute toxicity of organophosphorus (OP) pesticides and nerve agents is a result of inhibition of acetylcholinesterase (AChE), leading to accumulation of acetylcholine (ACh) at both muscarinic and nicotinic acetylcholine receptors; however, current pharmacotherapy focuses only on the muscarinic component, using the competitive muscarinic antagonist, atropine. The consequences of nicotinic overstimulation include paralysis of respiratory and other skeletal muscles, which is a major factor in the lethal effects of AChE inhibitors.

The paralysis produced by anticholinesterases is initially believed to result from depolarisation block of the muscle membrane due to potentiation and prolongation of the endplate potential as a result of the prolonged residence time of ACh in the synaptic cleft. During a period of repetitive (tetanic) stimulation, the force generated by the muscle rapidly fades as the muscle membrane becomes depolarised without a refractory period. This is known as depolarisation block because the muscle is unable to respond to new stimuli as it is already in a depolarised state [1–3]. Over a longer timescale, continuous stimulation by ACh causes the receptor to become insensitive to ACh binding [4, 5]. Failure of neuromuscular transmission during AChE inhibition is thus a combination of depolarisation block and receptor desensitisation. The interaction between these two processes is apparent after a prolonged period of AChE inhibition, when the ability of a neuromuscular preparation to sustain a tetanic contraction returns slightly. The mechanism of this adaptation is believed to be chronic desensitisation of the postsynaptic nicotinic receptors, which results in a reduction of the endplate potential amplitude and duration [1, 6]. The muscle membrane is then able to repolarise and undergo a refractory period, restoring contractile function. It has even been suggested that agents that accelerate desensitisation at the neuromuscular junction could be useful in the treatment of anticholinesterase poisoning [6].

Although the nicotinic effects can be treated indirectly, by the use of an oxime to reactivate inhibited acetylcholinesterase, each acetylcholinesterase-inhibitor complex differs in its susceptibility to reactivation by different oximes. Moreover, some complexes under a process called aging which renders them impossible to reactivate. This means that no one oxime will be effective against all of the OPs, making a generic therapy based on this mechanism unlikely [7].

In contrast, an appropriate antinicotinic drug (reviewed in [8]) should be able to treat the nicotinic effects of poisoning regardless of the OP involved, in the same way that the effectiveness of muscarinic antagonists is independent of the OP. The reason why nicotinic antagonists are not currently used [9] is largely due to the difficulties of administering a dose of competitive nicotinic neuromuscular blocker sufficient to antagonise the effects of excessive acetylcholine, but not so great that it paralyses the muscles [10].

An alternative approach would be to use a noncompetitive antagonist whose effects would not be overcome by increasing concentrations of acetylcholine. Certain bispyridinium compounds [11], including some oximes, have a beneficial effect in organophosphorus poisoning, which correlates with their ability to block the open ion channel of the nicotinic receptor [12]. Open channel block, a form of noncompetitive antagonism, appears to be an ideal way of mitigating the effects of excessive activation of nAChRs because the block is use-dependent: antagonism becomes greater as channel activation increases. This is the converse of what happens with a competitive antagonist.

We recently synthesised a novel bispyridinium compound which blocks the open ion channel of the muscle-type nicotinic receptor and we demonstrated that this noncompetitive antagonism, as well as reversing the neuromuscular blocking action of nerve agent *in vitro*, can protect animals against poisoning by nerve agents when used as part of a therapeutic drug combination [13, 14]. We have now synthesised a series of compounds based on this structure. We followed the approach to structure–activity relationship used previously by [15], who characterized the chain length dependence of potency of bis-quartenary N^+^ separated by polymethylenes of different chain lengths.

In this study, we demonstrate that the bis-pyridinium compounds interact not only with muscle-type nAChRs, but also with neuronal nAChRs containing only α and β subunits, and that the strength of the interactions with muscle nicotinic receptor, neuronal nicotinic receptor and acetylcholinesterase all depend on the length of the polymethylene chain linking the two pyridinium moieties. Understanding of structure-activity relationships will be valuable for optimising molecular structures for therapeutic use.

## Material and Methods

### Drugs and chemicals

**Fig 1:**
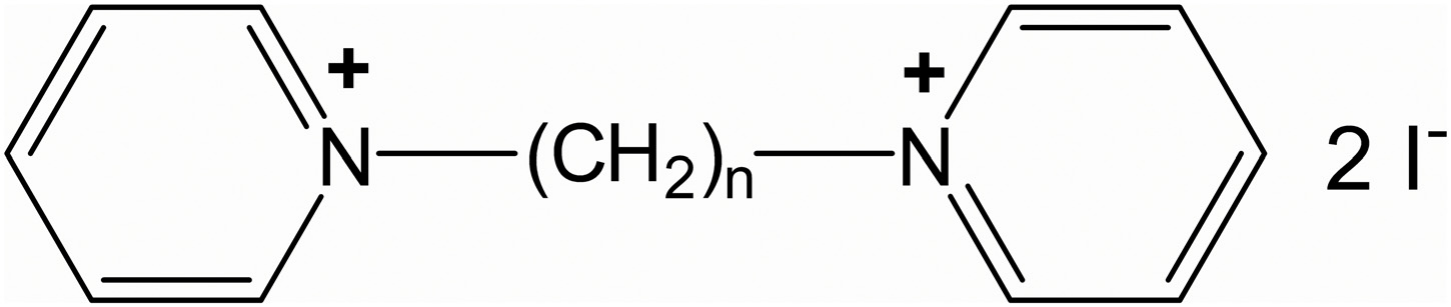
Molecular structure of the bispyridinium compounds tested.

Bispyridinium compounds (Fig 1), in which the polymethylene chain linking the two pyridinium rings was increased in length from C1 to C10, were synthesised as the diiodide salts at Dstl Porton Down and were >98% pure. These salts are easily accessible through quaternisation of two molar equivalents of pyridine with α,ω-diiodoalkanes [16] and many could be made in a short space of time for *in vitro* screening. The nature of the counterion is expected to have a negligible effect on the ion channel blocking activity as the latter depends on the nature of the cation. Unless otherwise noted, all proprietary drugs and chemicals were purchased from Sigma-Aldrich Ltd. (Poole, Dorset, UK).

### Cell culture

CN21 cells, derived from the TE671 human rhabdomyosarcoma cell line [17] by a stable transfection of the ε-subunit to express both the foetal (α1, γ, α1, ß1, δ) and adult human (α1, ε, α1, ß1, δ) muscle nicotinic receptor, were a gift from Dr David Beeson (Institute of Molecular Medicine, John Radcliffe Hospital, Oxford, UK) [18]. The cells were grown using standard cell culture techniques in Dulbecco’s Modified Eagle’s Medium (Sigma-Aldrich, UK) with 10% Foetal Bovine Serum (Invitrogen, UK), 50 units ml^-1^ penicillin, 50 μg ml^-1^ streptomycin, 2 mM L-glutamine (Sigma-Aldrich, UK) and 0.5 mg ml^-1^ geneticin (Invitrogen, UK) and grown in 150 cm^2^ cell culture flasks until approximately 70–80% confluent in a humidified atmosphere in an incubator at 36.5°C with 5% CO_2_. Cells were then harvested using 0.25% trypsin/ethylenediamine tetraacetic acid (EDTA) solution (Sigma-Aldrich, UK) in Ca^2+^/Mg^2+^-free phosphate buffered saline and were collected by centrifugation (100 g, 4 min). For maintenance, cells were re-plated into culture flasks at split ratios of 1:6–1:10. Cells were used in experiments between passages 2 and 8 following recovery from cryopreservation.

The human neuroblastoma cell line SH-SY5Y expresses several nicotinic receptor subunits (α3, α5, α7, β2 and β4), giving rise to multiple neuronal nicotinic receptor subtypes [19], which makes it a good model for investigating neuronal responses to nicotinic receptor activation. SH-SY5Y cells, generation 14, were obtained from the European Collection of Cell Cultures (ECACC, Salisbury, UK), scaled up and stored in ampoules at -135°C in the regular growth medium supplemented with 10% DMSO. Cells (generation 19–25) were grown in Minimum Essential Medium with Earle’s salts, GlutaMax-I, 10% Foetal Bovine Serum (all Invitrogen, Norway) and 0.1 mg ml^-1^ Gentamycin sulfate in poly-L-lysine coated 75 ml cell culture flasks at 36°C in a humidified atmosphere with 5% CO_2_. The medium was changed after one day to remove remnants of DMSO and again after 3–4 days. The cells were subdivided into new flasks with a split ratio of 1:10 every 6–8 days. The medium was removed and cells were harvested with the aid of 0.02% EDTA in phosphate buffered saline (i.e. without trypsin). Cells were detached with a tap on the flask and growth medium was added to the suspended cells which were collected by centrifugation (100 g, 3 min). For experiments, cells were plated at 1:10 dilution into poly-L-lysine coated 96-well black Cellbind flat transparent bottom plates (Corning, USA) and experiments were performed 72 h later with semiconfluent cultures.

### Nicotinic calcium response assay

CN21 cells were plated out onto clear-bottomed, black-walled, tissue culture treated 96-well plates (Corning Costar) at a density of ~20,000 cells per well in 100 μl of medium (so that they approached confluence after 24 h) for subsequent experimentation. The growth medium was removed, leaving the cells adhering to the bottom of the plate and 50 μl of a Calcium 4 assay kit dye (Fluo-4 acetoxymethylester, Molecular Devices FLIPR Calcium 4 assay kit, Molecular Devices, Union City, California, USA) in a HEPES-buffered balanced salt solution (NaCl 135 mM, KCl 5.4 mM, CaCl_2_ 1 mM, MgCl_2_ 1 mM, HEPES acid 5 mM, NaHCO_3_ 3.6 mM, D-glucose 10 mM, pH 7.4 with NaOH) was added and the cells were incubated in the dark for ~30 min. Cells were not washed prior to assay as the FLIPR Calcium 4 assay kit also contained a quenching dye to minimise fluorescence from extracellular de-esterified (fluorescent) Fluo-4. The dye loading solution also contained atropine (20 μM) to block the muscarinic response and pharmacologically isolate the nicotinic response to acetylcholine.

The test compounds were dissolved as stock solutions in HEPES-buffered solution on the day of the experiment. After the cells had been incubated with the dye, dilutions of this stock solution (12 μl) were added to each well on the cell plate to achieve the final desired concentrations. The first and last columns received a vehicle control so that the compound response could be compared to the vehicle at the beginning and end of each experiment to allow evaluation of time-dependent effects. Each compound was tested at 10 different concentrations in duplicate on each plate and each experiment was conducted 3 times.

For testing, plates were transferred to a FlexStation II fluorescence plate reader (Molecular Devices Limited, UK) and the fluorescence intensity was measured at ~1 s intervals prior to and after addition of 58 μl of 20 μM acetylcholine to achieve a final concentration in each well of ~10 μM, which gave approximately 80% of the maximal response (EC_80_). Excitation and emission wavelengths were set to 485 nm and 525 nm respectively, with a cut-off of 515 nm. All measurements were made at room temperature (~21°C). Agonist additions were made automatically using the plate reader’s in-built dispensing functions. Baseline fluorescence was measured for 20 s, followed by addition of acetylcholine. Further measurements of fluorescence for 80 s were then made to follow the agonist-induced response.

Responses were quantified as the maximum response expressed as a percentage of the average baseline values. For analysis, these data were then normalised to the mean vehicle control values for the wells in the corresponding row. Responses were fitted to a standard four-parameter logistic equation using GraphPad Prism version 4.00 for Windows (GraphPad Software, San Diego, California, USA) and a log IC_50_ and Hill slope with standard errors (SEM) were determined. For analysis of the lower potency compounds, curve fitting assumed a maximal inhibition equivalent to that obtained with the higher potency C7-C10 compounds where maximal inhibition could be fitted. This value was constrained in the analyses of the lower potency data allowing an estimation of the IC_50_.

SH-SY5Y cells grown in multiwell plates were loaded with fura-2 by incubating in growth medium with 2 μM fura-2 AM (Invitrogen, UK), 0.02% pluronic (Invitrogen) and 1 mM probenecid, the latter to inhibit fura-2 secretion [20]. Cells were incubated at 36°C for 45 min and then in the growth medium alone for ~35 min for de-esterification. The cells were then washed once with experimental buffer (140 mM NaCl, 3.5 mM KCl, 1 mM MgSO_4_, 2 mM CaCl_2_,1.2 mM Na_2_HPO_4_, 15 mM Tris-HCl, 5 mM glucose, 1 mM MgSO_4_) before adding experimental buffer with the drug tested to a total volume of 200 μl per well. Probenecid at 1 mM was added to all experimental buffers.

For testing, plates were transferred to a fluorescence plate reader equipped with injectors (Fluostar Optima, BMG Labtech, Germany). Nicotine was diluted to 250 μM in experimental buffer and filled in the injector. The plate was then placed in the plate reader set at a temperature of 32°C. The injection speed was 100 μl s^-1^, and the total injection volume was 50 μl, the final concentration then being 50 μM nicotine.

Fluorescence excitations were at 340 nm and 380 nm (10 nm bandwidths) and low pass emission at 505 nm, both in bottom reading mode (i.e. from underneath the plate). Time resolution was limited by the speed of filter wheel change to 1.5 s per sampling point. The total measurement time for each well was 30 s. The agonist was injected under the computerised control of the plate reader after 8 s (to determine first a baseline for each well individually) and the area under the curve for the last 15 s of emission was determined. The average background fluorescence from 340 nm and 380 nm excitation was determined from a plate with cultures of SH-SY5Y cells not loaded with fura-2 and the response in each well was taken as the change in the "340/380 ratio", i.e. the change in the ratio of emission from excitations at 340 nm and 380 nm in each well. Response calculations were automated off-line with an in-house programme for fura-2 and 96-well plates written in Origin (OriginLab, USA). IC_50_ values were determined in a similar way to the analysis of responses for CN21 cells by fitting the four-parameter logistic equation to the ratiometric responses using GraphPad Prism version 4.00.

### Acetylcholinesterase inhibition assay

Human erythrocyte ghosts served as the acetylcholinesterase source and were prepared from heparinized human blood. As confirmed by the Bavarian Medical Association, the investigation of blood samples was not considered a clinical study under German regulations and therefore did not require formal approval by an ethics committee.

Hemoglobin-free erythrocyte ghosts were prepared as described previously [21]. In brief, heparinized human blood was centrifuged (3000 x g, 10 min) and the plasma removed. Erythrocytes were washed with 2 volumes of phosphate buffer (3 times; 0.1 M, pH 7.4) and the packed erythrocytes were diluted in 20 volumes of hypotonic phosphate buffer (6.7 mM, pH 7.4) followed by centrifugation (50.000 x g, 30 min, 4°C). The supernatant was removed, the pellet re-suspended in hypotonic phosphate buffer and the procedure was repeated twice. Then, the pellet was re-suspended in phosphate buffer (0.1 M, pH 7.4), the virtually hemoglobin-free erythrocyte ghosts were concentrated by centrifugation at 100,000 x g (30 min, 4°C) and, finally, the AChE activity was adjusted to the original activity (i.e. 4–5 U/ml) by appropriate dilution with phosphate buffer (0.1 M, pH 7.4). Aliquots of the erythrocyte ghosts were stored at -80°C until use. In order to achieve a homogenous matrix for the kinetic studies, aliquots were homogenized on ice three-times for 5 sec with 30 sec intervals with a Sonoplus HD 2070 ultrasonic homogenator (Bandelin electronic, Berlin, Germany).

Acetylcholinesterase activities were measured spectrophotometrically with a liquid handling system (Freedom EVO, Tecan, Männedorf, Switzerland) connected to a microplate reader Safire^2^ (Tecan) with a modified Ellman assay [22, 23] using 96-well microplates and 0.45 mM acetylthiocholine iodide (Sigma-Aldrich, Deisenhofen, Germany) as substrate and 0.3 mM 5,5’-dithiobis(2-nitrobenzoic acid) (DTNB; Sigma) as a chromogen in 0.1 M phosphate buffer (pH 7.4, 37°C).

The inhibitory potency of the test compounds C1-C10 was assessed in duplicate by incubation of acetylcholinesterase with 10^−9^–10^−3^ M for 5 min before adding substrate to determine enzyme activity. Acetylcholinesterase activities were referred to activity of native acetylcholinesterase and were expressed as percentage of control. IC_50_ values were determined in a similar way to the analysis of responses for CN21 and SH-SY5Y cells by fitting a four parameter logistic equation (GraphPad Prism 4.00).

## Results

### Nicotinic response in CN21 cells

When CN21 cells were exposed to acetylcholine in the presence of atropine (20 μM) to block the muscarinic response, an influx of Ca^2+^ could be measured using the Ca^2+^-sensitive fluorescent dye Fluo-4. This response peaked in less than 10 s and could be completely blocked by the addition of the nicotinic antagonist d-tubocurarine (50 μM) [13]. When KCl at concentrations of 10–100 mM was used to depolarise the cells without addition of acetylcholine, no response was elicited (data not shown), confirming that the response was due to activation of nAChRs.

**Fig 2:**
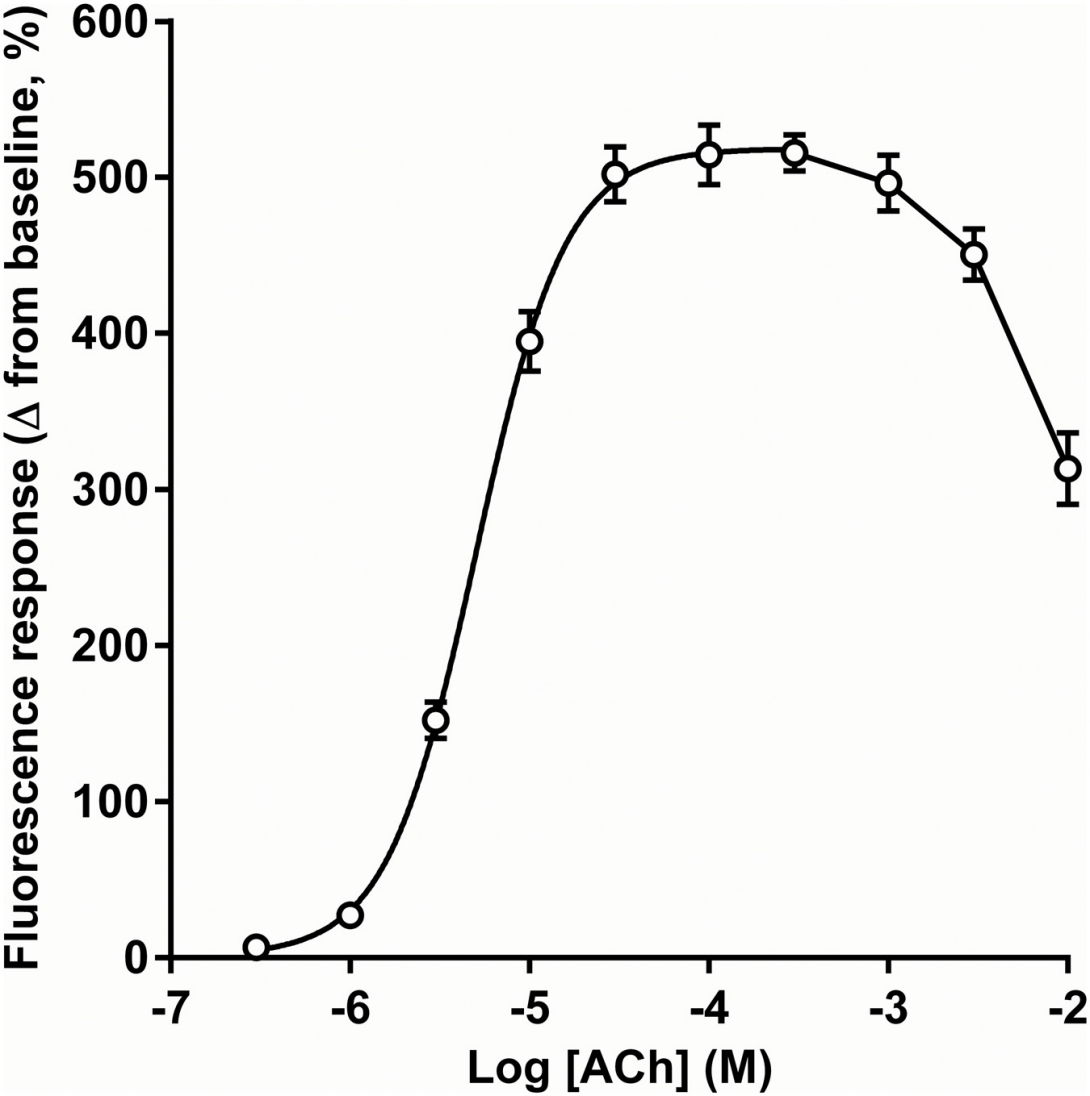
Concentration-response relationship for ACh in CN21 cells. This graph shows the effect of ACh (in the presence of 20 μM atropine) on the fluorescence response measured in CN21 cells. Data are displayed as the mean ± SEM of four replicates on a single plate. The sigmoidal concentration-response curve was fitted for concentrations between 3 x 10^−7^ and 3 x 10^−4^ M. Best fit values: EC_50_ = 5.0 × 10^-6^ M; Log EC_50_ = -5.3; Hill slope = 1.7

The concentration-dependence of the nicotinic response is shown in Fig 2. The response increased rapidly from a negligible change in fluorescence at 0.3 μM acetylcholine to a plateau of the maximum response from 30 to 300 μM, declined at higher concentrations. To enable a parametric fit of the data shown in Fig 2, concentrations above 300 μM were removed from the analysis and a sigmoidal concentration-response curve was fitted to the data, revealing a Hill slope greater than 1. Using these data, an agonist concentration of 10 μM ACh, which represented an approximate EC_80_ of the response, was selected for subsequent experiments using the test compounds.

### Effect of the test compounds on CN21 cells

**Fig 3:**
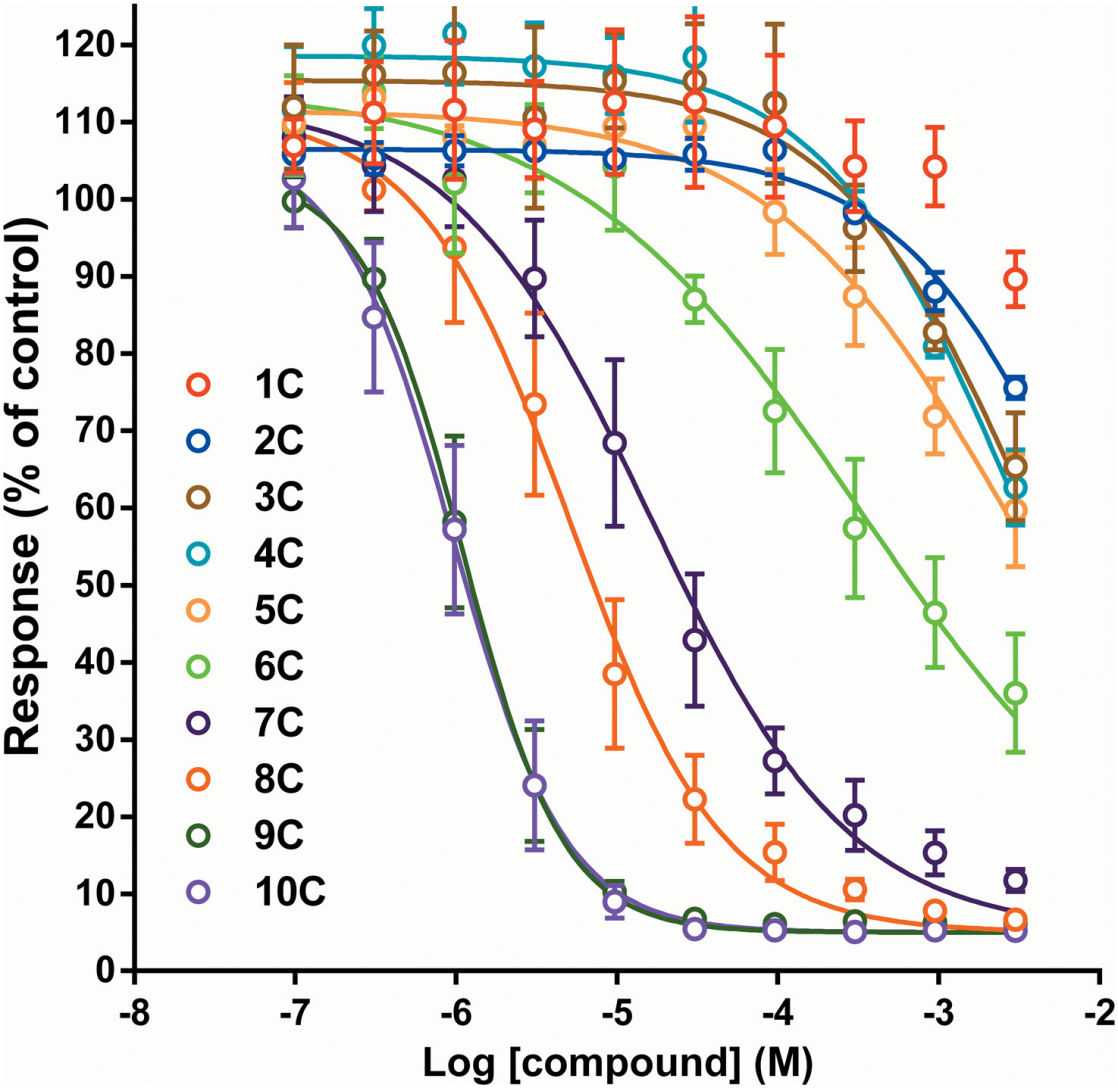
Influence of linker length on the effect of the bispyridinium compounds in CN21 cells. The Ca^2+^ response to ACh (10 μM) was measured in CN21 cells using a FlexStation plate reader in the presence of different concentrations of the test compounds. Data are displayed as the mean ± SEM of three experiments performed in duplicate on each plate. Curves were fitted with a standard four-parameter logistic equation (see text). The fit did not converge for the C1 compound (MB775).

Each compound was tested on CN21 cells at 10 concentrations, decreasing by half-log steps from 3 mM. All of the compounds produced a concentration-dependent inhibition of the nicotinic response (Fig 3). The inhibitory potency of the series of compounds increased with longer linker length (Table 1). The C1 to C5 compounds generally had a limited effect on the nicotinic response at the concentrations tested here, but the C6 to C10 compounds all produced a potent block of the response (Fig 3). The C9 and C10 compounds had almost identical profiles of activity in this assay. All the Hill slope values (Table 1) were less negative than -1, except for the C9 and C10 compounds which produced steeper slopes (closer to -1.5).

**Table 1:** Inhibition of nicotinic responses in CN21 and SH-SY5Y cells.

		CN21		SH-SY5Y	
Test compound	n	Log IC_50_ (M)	Hill slope	Log IC_50_ (M)	Hill slope
**MB775**	1	DNC	DNC	-	-
**MB520**	2	-2.06 ± 0.04	-0.75 ± 0.06	-	-
**MB408**	3	-2.37 ± 0 26	-0.73 ± 0.06	-	-
**MB444**	4	-2.51 ± 0.15	-0.72 ± 0.05	-3.27 ± 0.04	-1.81 ± 0.26
**MB442**	5	-2.56 ± 0.16	-0.60 ± 0.13	-4.01 ± 0.04	-2.10 ± 0.40
**MB776**	6	-3.50 ± 0.32	-0.50 ± 0.02	-4.54 ± 0.04	-1.88 ± 0.24
**MB777**	7	-4.81 ± 0.23	-0.72 ± 0.01	-4.43 ± 0.05	-1.80 ± 0.30
**MB505**	8	-5.30 ± 0.20	-0.99 ± 0.07	-4.76 ± 0.03	-1.74 ± 0.22
**MB778**	9	-5.94 ± 0.11	-1.56 ± 0.11	-5.38 ± 0.04	-1.66 ± 0.20
**MB779**	10	-6.05 ± 0.17	-1.34 ± 0.14	-5.78 ± 0.03	-1.28 ± 0.09

Log IC_50_ values for inhibition of nicotinic responses in CN21 and SH-SY5Y cells by the test compounds. Data show mean ± SEM. All values were obtained by best-fit of the Hill-equation as described in the methods section. DNC = fit did not converge.

### Nicotinic response in SH-SY5Y cells

In preliminary experiments (not shown), SH-SY5Y cells cultured on poly-L-lysine coated glass coverslips were exposed to nicotine, 10–100 μM. The single cell measurements showed responses with an increase in intracellular Ca^2+^ which peaked in 15–40 s depending on the agonist concentration and the size of the response.

**Fig 4:**
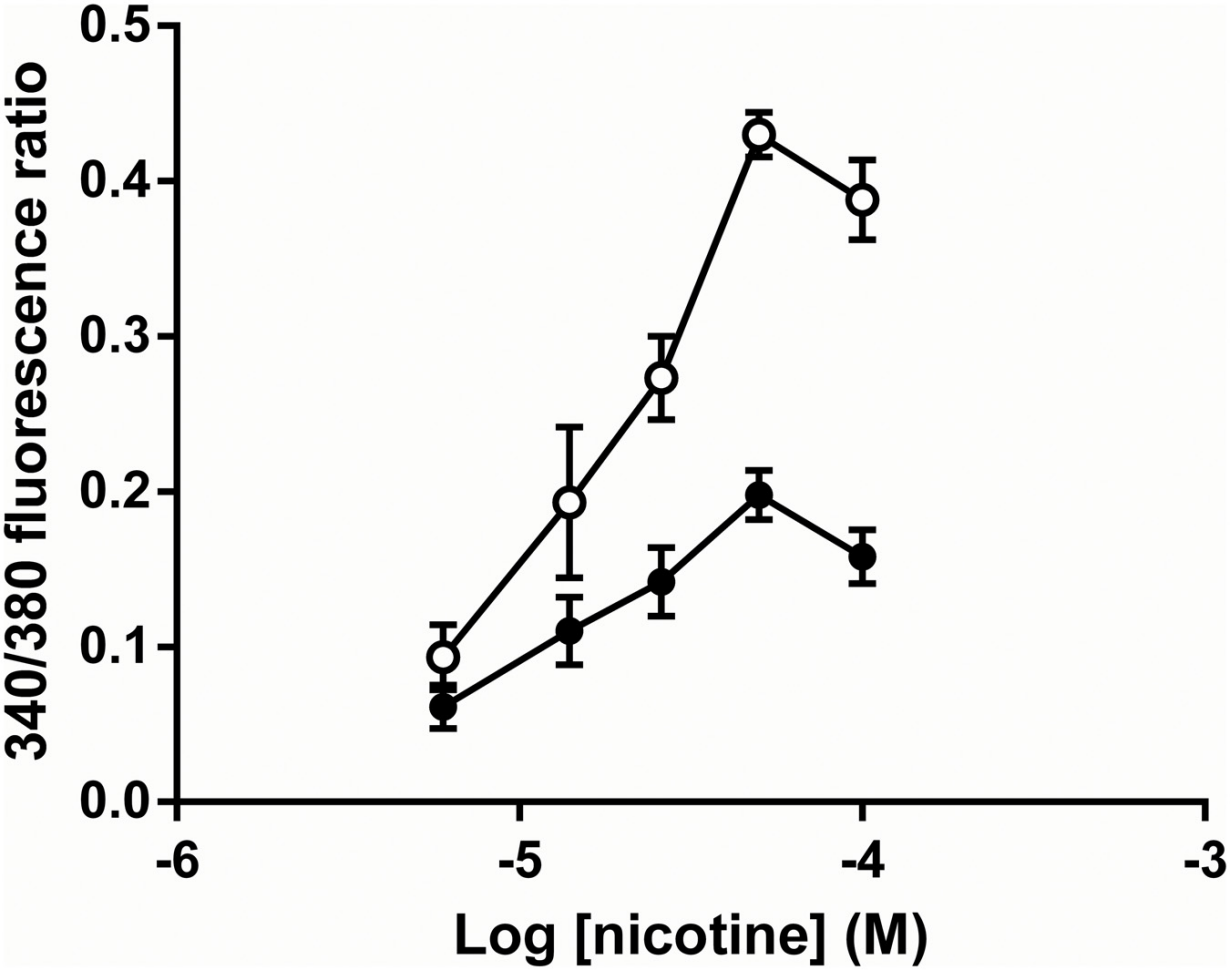
Concentration-response relationship for nicotine-evoked calcium responses in SH-SY5Y cells. The graph shows the inhibitory effect of the C10 bispyridinium compound MB779. Open circles, nicotine alone; closed circles, nicotine + MB 779 (2 μM). Data are displayed as the mean ± SEM of two experiments with eight replicates on each plate.

For the remainder of the experiments, SH-SY5Y cells were cultured in 96-well plates. Fig 4 shows the concentration-response relationship for nicotine and the effect of one of the test compounds (MB779, C10) on the concentration-response curve. The responses were completely blocked by mecamylamine, a selective neuronal nicotinic receptor inhibitor, and by d-tubocurarine, with EC_50_ values of 0.025 μM and 0.66 μM, respectively. A concentration of 50 μM nicotine, corresponding to the peak of the dose response curve, was selected for subsequent experiments comparing the effects of the test compounds on nicotinic receptor-mediated responses in SH-SY5Y cells.

### Effect of the test compounds on SH-SY5Y cells

**Fig 5:**
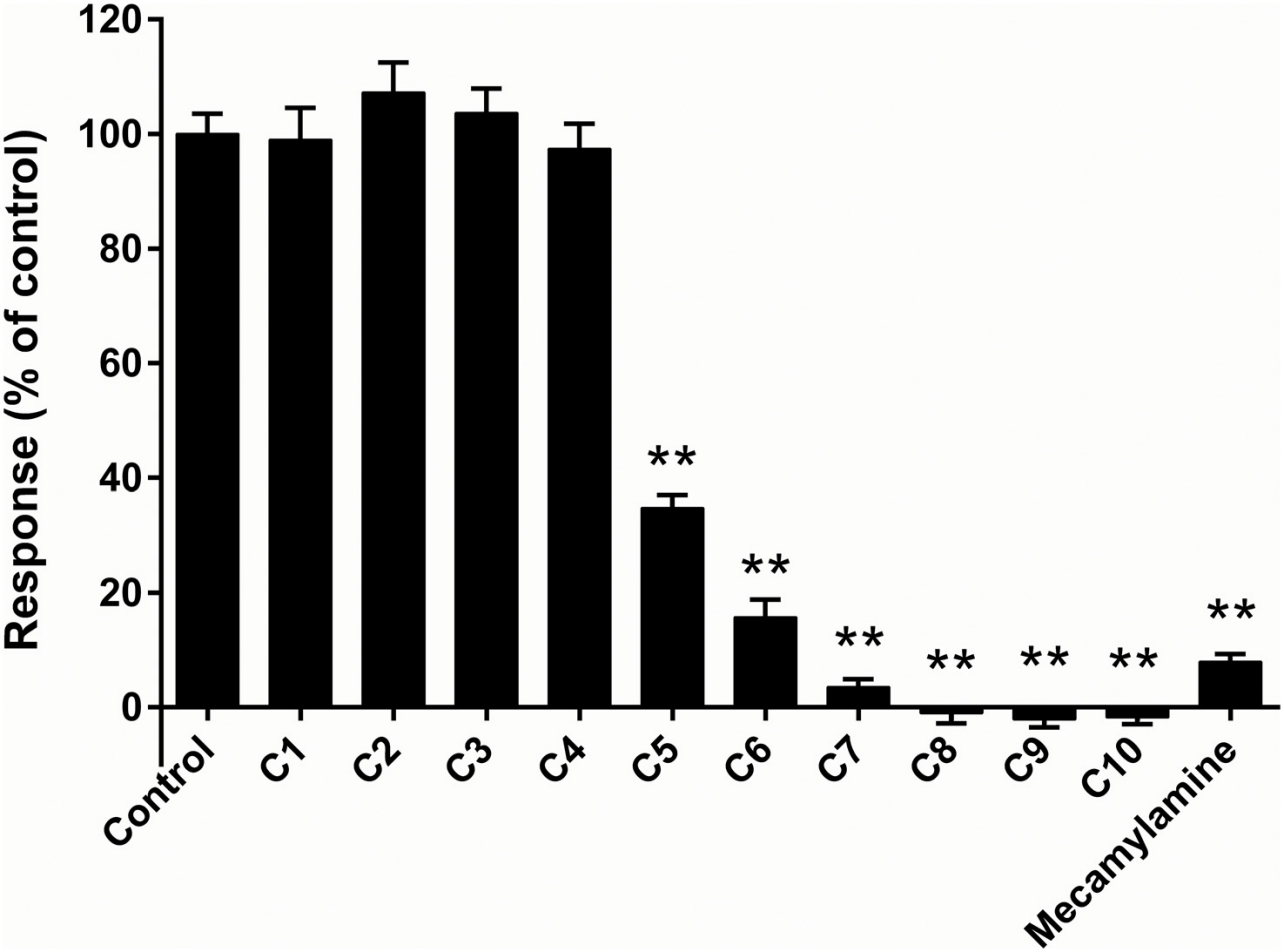
Inhibition of nicotinic responses in SH-SY5Y cells. Ca^2+^-responses were induced by nicotine (50 μM) and the test compounds were all applied at a concentration of 100 μM. Data are displayed as the mean ± SEM of two experiments with eight replicates on each plate. ** significant difference from control (p<0.01, ANOVA with Dunnet’s post hoc test).

**Fig 6:**
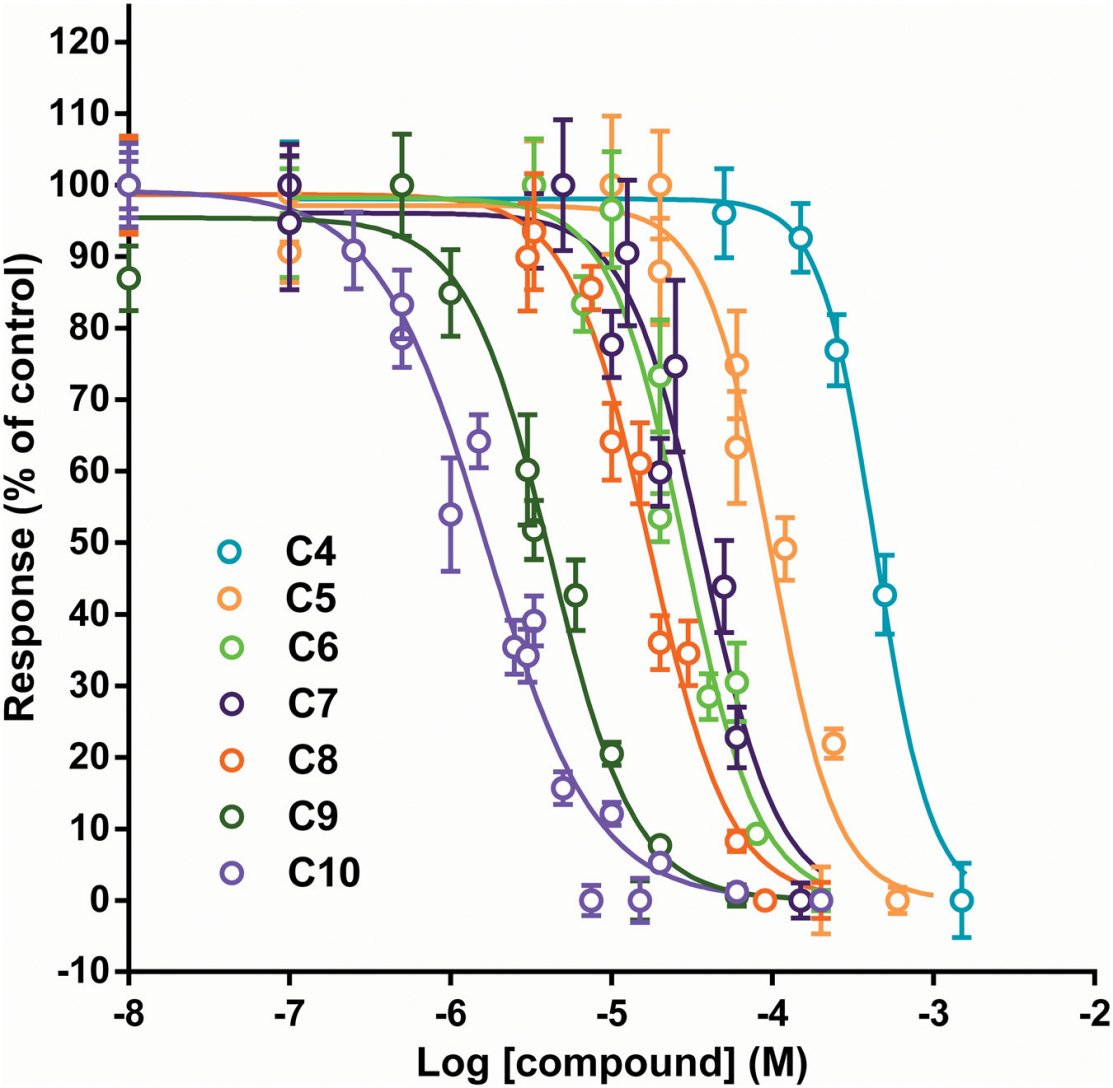
Influence of linker length on the effect of the test compounds in SH-SY5Y cells. The Ca^2+^ response to nicotine (50 μM) was measured in SH-SY5Y cells using a Fluostar Optima plate reader. Data are displayed as the mean ± SEM of two experiments with eight replicates on each plate. Curves were fitted were fitted with a standard four parameter logistic equation (see text).

Each compound was tested on SH-SY5Y cells at a range of concentrations similar to those tested on CN21 cells. When tested at 100 μM, the C1-C4 compounds had no significant effect on the responses (Fig 5) and therefore only C4-C10 were characterised at a larger range of concentrations. All of these compounds produced a concentration-dependent inhibition of the nicotinic response in SH-SY5Y cells (Fig 6). The antinicotinic inhibitory potency of the series of compounds increased with longer linker length (Table 1). In contrast to the results in CN21 cells, all the Hill slope values (Table 1) were more negative than -1.

In separate experiments using potassium depolarisation (not shown), it was verified that the inhibitors had no effect on Ca^2+^ responses from activation of L-type calcium channels.

### Inhibition of acetylcholinesterase

The inhibitory potency of the series of compounds to acetylcholinesterase also increased with longer linker length (Table 2). The C1 to C4 compounds had a limited effect on the enzyme activity at concentrations up to 1 mM, but the C5 to C10 compounds inhibited acetylcholinesterase activity with increasing potency, with the C9 and C10 compounds having IC_50_ values less than 1 μM. Table 2 also shows previously-published LD_50_ data for some of the compounds [24, 25] and, for comparison, some common oximes [26].

**Table 2:** Inactivation of human acetylcholinesterase and intravenous toxicities in mice.

Compound	n	AChE IC_50_ (μM)	Log AChE IC_50_ (M)	LD_50_ iv mice (mg kg^-1^)
***Bispyridinium series*** ^*a*^				
**MB775**	1	>1000	>-3	-
**MB520**	2	>1000	>-3	225
**MB408**	3	>1000	>-3	180
**MB444**	4	>1000	>-3	55
**MB442**	5	700	-3.15	32
**MB776**	6	70	-4.15	26
**MB777**	7	20	-4.70	-
**MB505**	8	1.4	-5.85	-
**MB778**	9	0.2	-6.70	-
**MB779**	10	0.5	-6.30	15
***Common oximes*** ^*b*^				
**HI-6**	-	-	-	178
**P2S**	-	-	-	116
**2-PAMCl**	-	-	-	93
**Obidoxime**	-	-	-	70

IC_50_ values for inactivation of human acetylcholinesterase and intravenous toxicities in mice for the test compounds and some oximes commonly used to treat nerve agent poisoning.

^a^ LD_50_ data for dibromide salt variants of the MB compounds taken from [24] and [25].

^b^ Averaged LD_50_ data from [26].

## Discussion

The results of this study demonstrate that bispyridinium compounds inhibit nicotinic receptor-mediated calcium responses in CN21 and SH-SY5Y cells and show a similar structure-activity relationship in both cell lines. There was a profound effect of altering the polymethylene linker length between the pyridine rings.

### Mechanism of Ca^2+^ responses in CN21 and SH-SY5Y cells

The Ca^2+^ that produces the increase in fluorescence in the CN21 cell assay appears to be entering the cell through the cation-selective nicotinic ion channel [27]. Firstly, atropine completely blocked the muscarinic component of the ACh-induced Ca^2+^ response in these cells, the remaining response being abolished by the nicotinic antagonist d-tubocurarine. The response is therefore not due to muscarinic receptor-induced Ca^2+^ release from intracellular stores [13]. Secondly, depolarisation of the cells with KCl failed to elicit a calcium response, indicating that they do not express voltage-gated Ca^2+^ channels (VGCCs). This conclusion is in agreement with both functional [28] and genetic [29] assessments of TE671 cells (the cell line from which CN21 was derived), in which no VGCCs were found. [28] also showed that omission of Ca^2+^ from the extracellular solution abolished the response to ACh and that neither treatment with K^+^ nor the L-type Ca^2+^ channel activator (-)BayK8644 produced an increase in intracellular Ca^2+^. Although it is conceivable that Ca^2+^-induced Ca^2+^ release would further increase the calcium response, it can be surmised from this evidence that the response in CN21 cells depends on Ca^2+^ influx through the nicotinic ion channel when activated by ACh.

In SH-SY5Y cells, the nicotinic receptor-activated Ca^2+^ influx is due to influx through Ca^2+^ permeable nAChRs as well as activation of a number of parallel pathways [30], in particular due to the presence of voltage-activated Ca^2+^ channels [31] and Ca^2+^-mediated Ca^2+^ release. Nevertheless, since the specific nicotinic receptor inhibitors mecamylamine and d-tubocurarine completely inhibit the responses, the different sources of cytosolic Ca^2+^ rise are secondary to nicotinic receptor activation. The effect of the bispyridinium compounds on nicotine-activated Ca^2+^ responses was therefore taken as the inhibitory effect on nAChRs.

### Inhibition of nicotinic receptor mediated responses

**Fig 7:**
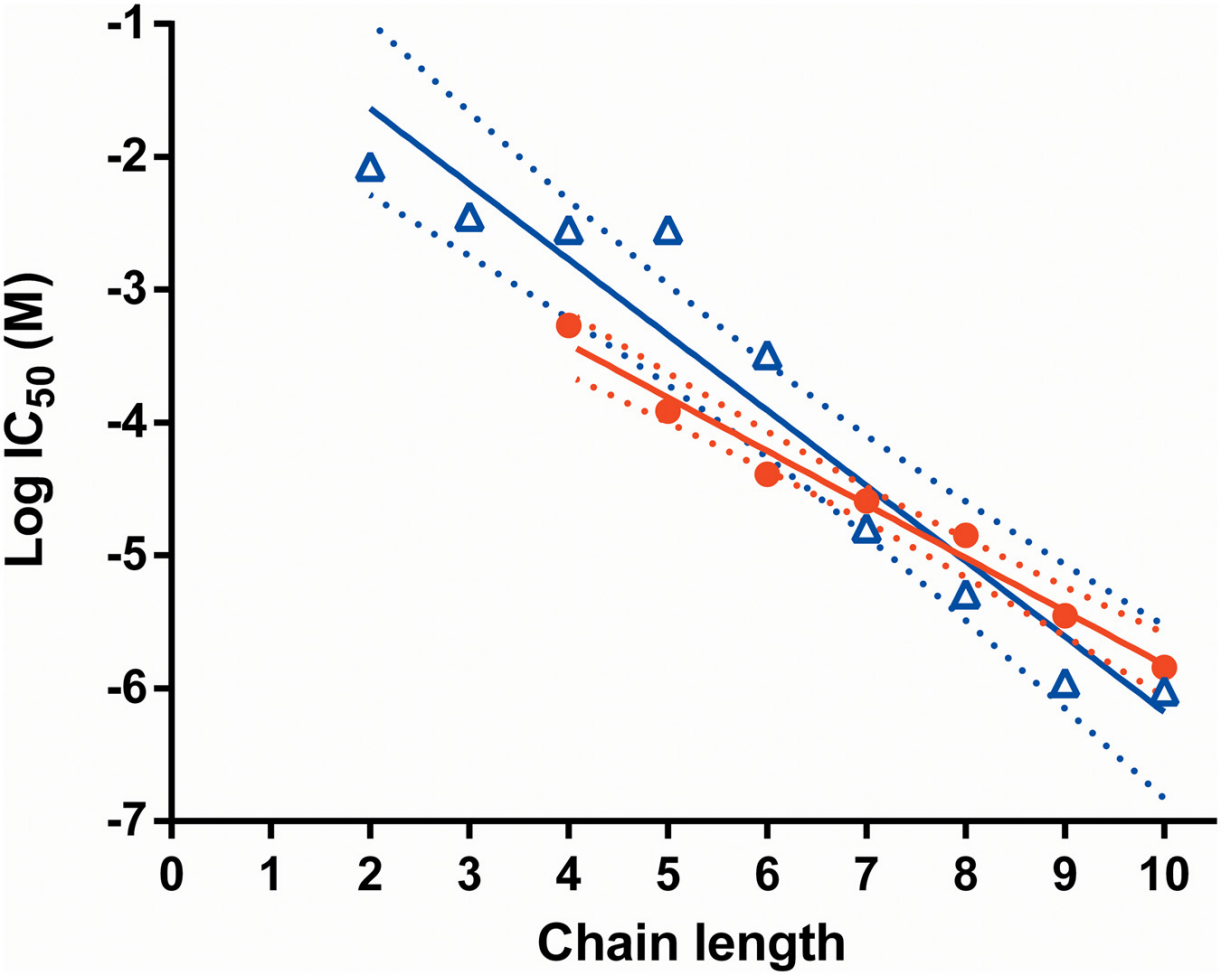
Relationship between polymethylene linker chain length and log IC_50_. Data are shown for CN21 (open blue triangles) and SH-SY5Y (closed red circles) cells. Solid lines show linear regression fits of the data for the two data sets: CN21, y = -0.575x - 0.450, r^2^ = 0.936; SHSY5Y, y = -0.402x - 1.799, r^2^ = 0.980. Broken lines show the 95% confidence intervals of the fits.

The inhibitions of nicotinic responses produced by the compounds in the two cell types are compared in Table 1 and Fig 7. In both cell types, the inhibitory potency (indicated by IC_50_) of the series increased with longer linker length. The clear relationship between increasing chain length and increasing potency suggests that either the relative positions of the charged nitrogen groups or the size of the molecule are the most important factors in determining the level of nicotinic antagonism for this series of compounds. This relationship was more marked for CN21 cells than for SH-SY5Y (slopes of -0.575 and -0.402, respectively), but the IC_50_ values in both cell types were similar for each compound, particularly for those with linker lengths greater than C6. In CN21, but not in SHSY5Y, there was a change in the slope of the relationship between C5 and C6. Our series of compounds has similarities to the polymethylene bistrimethylammonium series (Me_3_N^+^(CH_2_)_n_N^+^Me_3_ 2X^-^) studied by [15], who also commented on an abrupt change in the slope of the relationship for neuromuscular block around their C6 compound and suggested that this may indicate different pharmacological actions. They also found that, as with our compounds, the neuromuscular blocking potency increased up to C10.

For CN21 cells, the Hill coefficient was less negative than -1 for the C1 to C7 compounds, but then became more negative for longer linker lengths (C8 to C10). In contrast, in SH-SY5Y cells, it was closer to -2 for many of the compounds, becoming less negative as the linker length increased. It is conceivable that the difference in Hill coefficient between the two cell lines reflects the different subunit composition of the receptors in the two cell lines. CN21 cells express both foetal (α1-γ-α1-ß1-δ) and adult (α1-ε-α1-ß1-δ) human muscle nAChRs [13, 18], while the SH-SY5Y cell line expresses several nicotinic receptor subunits (α3, α5, α7, β2 and β4), giving rise to multiple nicotinic receptor subtypes [19].

Some bispyridinium compounds have been shown to block the open channel of the nicotinic receptor, a form of noncompetitive inhibition [12, 13], although a number of different mechanisms of action could be responsible for the effects of the test compounds in this study. Compounds which inhibited the response could be competitive or non-competitive antagonists at the nicotinic receptor, or agonists producing desensitisation or partial activation. Due to the limitations of the Ca^2+^ mobilisation fluorescence assay [32], it was not possible to confirm competitive or non-competitive mechanisms of action of these compounds [13]. Therefore, elucidation of the exact mechanism of the block will require further experiments, for example using a single-channel assay [13].

In a previous study, we showed that MB327, a 4-*tert*-butyl substituted analogue of MB408, produced open-channel block characterised by rapid, flickering bursts of openings and a reduction in the mean open time [13]. The effect of this would be not only to reduce the amplitude of the endplate current (and hence of the endplate potential), but also to shorten its duration. This would be expected to counteract the prolongation of the endplate potential by an AChE inhibitor and the subsequent depolarisation block during repetitive stimulation. Although effects on receptor desensitisation have not been investigated and cannot be ruled out, the alleviation of depolarisation block is consistent with the ability of MB327 to reverse the tetanic fade produced by soman and to restore the shape of the tetanus in guinea-pig phrenic-nerve hemidiaphragm preparations [13]. Furthermore, the di(methanesulfonate) salt of this compound has been shown to increase protection against tabun, sarin and soman when used as part of a combination therapy in guinea-pigs [13, 14]

### Inhibition of acetylcholinesterase

As well as inhibiting nicotinic receptor-induced Ca^2+^ responses, the test compounds also inhibited human acetylcholinesterase activity *in vitro*. As with the inhibition of nAChRs, the potency increased with increasing linker length, again suggesting that either the relative positions of the charged nitrogen groups or the size of the molecule are important factors in determining the level of activity for this action in these compounds. A similar relationship between anticholinesterase potency and chain length was reported by [15] for their polymethylene bistrimethylammonium compounds.

### Toxicity of the compounds

Toxicity data have been published for dibromide salts of some of these bispyridinium compounds [24, 25]: these show that the LD_50_ in mice decreases as the linker chain length increases (Table 2). The increasing potency of inhibition of both the nicotinic response and acetylcholinesterase activity produced by increasing the chain length of these compounds could be at least partly responsible for increasing toxicity through the series. The LD_50_ values indicate that the shorter chain analogues (C2 and C3) have toxicities similar to those of some of the oximes used to treat organophosphorus poisoning.

### Clinical and therapeutic considerations

Some of the compounds evaluated in this study were weak inhibitors of the nicotinic response and failed to block the response completely over the concentration range examined here. In CN21, but not in SH-SY5Y, the concentration-response relationships for these compounds tended to have shallower Hill slopes than those exhibited by the potent blockers. A compound with a shallow Hill slope could have more utility as a medical therapy against the nicotinic effects of organophosphorus poisoning, because it may have a larger therapeutic window before the compound itself becomes toxic through overwhelming block of nicotinic receptor function. Indeed, the 4-*tert*-butyl substituted analogue MB327 (with a C3 linker), which we previously showed to produce recovery of neuromuscular function following soman poisoning *in vitro* and to protect against nerve agent poisoning *in vivo* [13, 14], had a log IC_50_ [M] of -2.86 ± 0.16 (mean ± SEM) in the CN21 calcium response assay, with a Hill slope of -0.89 ± 0.10 [13]. More recently, we have demonstrated similar protection against soman poisoning using MB442, the C5 linker compound described in this study (unpublished data). Compounds that exhibit a steep concentration-response curve may require more careful titration of the therapeutic dose.

The similarity of the IC_50_ values in CN21 and SH-SY5Y assays suggests that the inhibition is not specific for particular subtypes of the nicotinic receptor, in particular since SH-SY5Y contains a number of channels with different stoichiometry of α and β subunits. This raises the possibility that some of these compounds could be used to treat the nicotinic effects of organophosphorus poisoning in the central nervous system if they could be delivered into the brain. Like oximes, these quaternary compounds would not be expected to cross the blood-brain barrier in significant quantities; however, there have been some recent studies on improving the penetration of oximes into the brain [33–35] and it is likely that these approaches could also be applied to these antinicotinic compounds.

## Conclusions

This study has demonstrated the ability of bispyridinium compounds to inhibit both muscle and neuronal nAChRs and has shown a clear structure-activity relationship based on the linker length between the two pyridinium moieties. The toxicities of the shorter chain analogues compare favourably to those for oximes used to treat organophosphorus poisoning [24, 25]. Furthermore, previous work has shown that a 4-*tert*-butyl substituted analogue of the C3 compound protected against nerve agent poisoning in guinea-pigs [13, 14]. Together, these results indicate that non-oxime bispyridinium compounds may be considered as promising candidates for the treatment of the nicotinic effects of organophosphorus poisoning, and that structure-activity relationships can be used to optimise molecular structures for therapeutic use.
